# Unraveling the controversy between fasting and nonfasting lipid testing in a normal population: a systematic review and meta-analysis of 244,665 participants

**DOI:** 10.1186/s12944-024-02169-y

**Published:** 2024-06-27

**Authors:** Ahmed B. Zaid, Samah M. Awad, Mona G El-Abd, Sara A. Saied, Shimaa K. Almahdy, AbdulRahman A Saied, Alshimaa M. Elmalawany, Hind S. AboShabaan, Helmy S. Saleh

**Affiliations:** 1 Department of Clinical Pathology, National Liver Institute, Menoufia University, Shibin Elkom, 32511 Egypt; 2 Department of Clinical Microbiology, National Liver Institute, Menoufia University, Shibin Elkom, 32511 Egypt; 3 Department of Hepatology and Gastroenterology, National Liver Institute, Menoufia University, Shibin Elkom, 32511 Egypt; 4 Ministry of Tourism and Antiquities, Aswan Office, Aswan, 81511 Egypt; 5 Department of Microbiology, Animal Health Research Institute, Shibin Elkom, 32511 Egypt; 6 Department of Clinical Pathology, National Liver Institute Hospital, Menoufia Univerisity, Shebin Elkoom, Egypt

**Keywords:** Fasting, Nonfasting, Lipid profile testing, Prediction, Healthy population

## Abstract

**Background:**

The final decision to fast or not fast for routine lipid profile examination in a standard, healthy population is unclear. Whereas the United States and European protocols state that fasting for regular lipid analysis is unnecessary, the North American and Chinese guidelines still recommend fasting before routine lipid testing.

**Aim:**

This study aimed to unravel the contradiction between the different protocols of lipid profile testing worldwide and clarify the effect of diet on lipid profile testing only in a regular, healthy population.

**Methods:**

A literature search was conducted through May 2024. The analyses included studies performed from the date 2000 until now because the contradiction of guidelines for lipid profile testing appeared for the first time in this period. A planned internal validity evaluation was performed using the National Institute of Health (NIH) quality measurement tools for observational cohort, case‒control, controlled interventional, and cross-sectional studies. The data were synthesized according to RevMan 5.3.

**Results:**

Eight studies with a total of 244,665 participants were included. The standardized mean difference in cholesterol in six studies showed significant differences in overall effect among fasting and nonfasting states (*P* < 0.00001), as did high-density lipoprotein cholesterol (*P* < 0.00001). At the same time, with respect to triglycerides and low-density lipoprotein cholesterol, there were notable variations in the overall effect between the fasted and nonfasted states (*P* < 0.00001 and *P* ≤ 0.001, respectively).

**Conclusions:**

This meta-analysis concluded that fasting for lipid profile testing is preferred as a conservative model to reduce variability and increase consistency in patients’ metabolic status when sampling for lipid testing.

**Supplementary Information:**

The online version contains supplementary material available at 10.1186/s12944-024-02169-y.

## Introduction

Examining fasting blood lipid levels can offer valuable information about the effects of different diets and metabolic processes. However, it is important to consider whether these levels accurately reflect the impact of individual foods or meals consumed throughout the day. For 24 h, the human body remains in a state of nonfasting and absorptive state for more than 18 h [1]. In a study conducted by Acevedo-Fani and Singh [2], the processes of digesting, absorbing, incorporating into the circulatory system, and clearing lipids from different foods and meals were influenced by a range of factors that can be classified into two categories: modifiable and unmodifiable. Factors that cannot be changed include diseases, genetic history, sex, age, and menstrual status; however, lifestyle choices such as engaging in regular exercise, smoking cigarettes, consuming alcoholic beverages, taking prescription drugs, and making specific food choices are regarded as factors that can be modified. Various factors influence the body’s ability to process lipids [3]. In individuals with average weight and those who are obese, consuming a single meal with a higher total fat content leads to an increase in the postprandial response of chylomicron triglycerides [4].

**Fig. 1:**
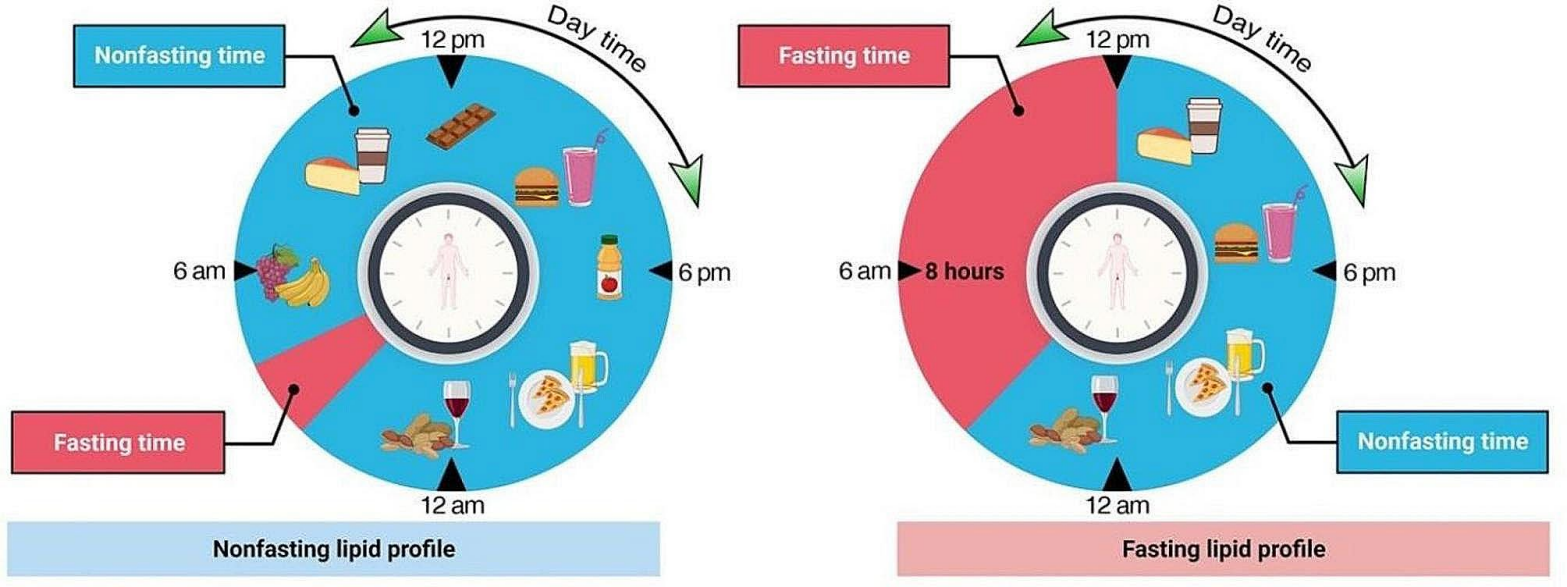
Fasting and nonfasting lipid testing protocols. Fasting for eight hours is enough to reduce variability and increase consistency in patients’ metabolic status at the time of sampling for lipid testing

Although humans typically do not fast or consume less fat regularly, it was previously believed that blood samples for lipid assessment should be taken after 8–12 h of fasting. This was based on the changes in serum triglycerides during a fat tolerance test. Furthermore, fasting helps to prevent lipemic serum and ensures accurate measurement of low-density lipoprotein (LDL) levels using the commonly used Friedewald’s formula in the laboratory [5]. Nonfasting samples have numerous clear advantages:

1. Staying away from the difficulty of prolonged fasting and early morning sampling.
2. Minimizing the risk of hypoglycemia in diabetic patients.
3. A nonfasting state is better for cardiovascular risk prediction, according to the guidelines in many countries [6, 7].

Research has demonstrated the strongest correlation between peak triglyceride levels measured four hours after meals and a cardiovascular event [8, 9]. Furthermore, there is evidence suggesting a correlation between insulin resistance and lipid or lipoprotein levels after a meal [10]. In addition, postmeal triglyceride levels that are greater than average and lower levels of high-density lipoprotein (HDL) cholesterol can be strong indicators of insulin resistance [11]. Community-based studies have shown that consuming food and following nonfasting routines for routine lipid testing have resulted in minimal changes in lipid profiles that are not clinically significant [6, 7, 11–15].

Major prospective trials have reported significant changes in various lipid parameters. The changes recorded were as follows: triglycerides increased by 0.3 mmol/L (26 milligrammes/dL), total cholesterol decreased by 0.2 mmol/L (8 milligrammes/dL), HDL cholesterol decreased by 0.1 mmol/L (4 milligrammes/dL), LDL cholesterol decreased by 0.2 mmol/L (8 milligrammes/dL), the calculated remnant cholesterol increased by 0.2 mmol/L (8 milligrammes/dL), and the estimated non-HDL cholesterol increased by 0.2 mmol/L (8 milligrammes/dL). The study revealed that the levels of HDL cholesterol, apolipoprotein A1, apolipoprotein B, and lipoprotein(a) remained unaffected by whether the participants were fasting or non-fasting.

Furthermore, the capacity to predict cardiovascular diseases using both nonfasting and fasting concentrations is similar [6, 7, 12]. Fasting lipid testing is recommended if triglyceride levels exceed 440 mg/dL when not fasting [7, 16].

The American Heart Association’s (ACC/AHA) recommendations do not call for fasting to estimate the risk of atherosclerotic cardiovascular disease [17]. It is important to remember that performing a fasting lipid profile to evaluate LDL cholesterol levels is recommended. This is especially important for individuals with non-HDL cholesterol levels below 5.7 millimol/L (220 milligrams/dL) or triglyceride levels above 5.7 millimol/L (500 milligrams/dL). These lipid profiles can be used as possible indicators for inherited and secondary factors contributing to hypertrophy [7]. This study sought to consolidate the results of previous smaller studies into a comprehensive meta-analysis. The goal of this study was to investigate the potential impact of fasting, nonfasting, or both on lipid profile testing in the general population. This study represents a groundbreaking meta-analysis involving a substantial sample size of 244,665 participants. It aims to shed light on the global controversy surrounding this subject.

## Resources and procedures

### Methods

The current systematic review is reported under the guidelines set by the Preferred Reporting Items for Systematic Reviews and Meta-Analyses (PRISMA) checklist, which is widely recognized as the standard for reporting systematic reviews [18]. This systematic review’s methodology adheres to the most recent edition of the Cochrane Handbook for Systematic Reviews of Interventions [19]. Additionally, it has been registered on Prospero with the number CRD42022376871.

### Data sources

This study thoroughly searched various online databases, such as Medline (via PubMed), Scopus, Web of Science, Cochrane Library, Virtual Health Library (VHL), and Global Index Medicine (GHL), as well as the references of the included studies. Additionally, the study explored related articles up to May 2024.

This study consists of studies performed from 2000 until now because the contradiction of guidelines for lipid profile testing appears for the first time in this period. Broad search filters were applied to find all the studies by using the following search strategy: (“Lipids” OR (“fatty acids”) OR “Ceroids” OR “Fats” OR “Glycerides” OR “Glycolipids” OR” Lipoproteins” OR “Lipopolysaccharides”) AND (“Fast* “OR” Fasting” OR (“Hunger Strikes”) OR (“Intermittent fasting”) OR (“Time-Restricted Feeding”)) AND (“Postprandial Periods”) OR “non-Fast$” OR” nonFast$” OR” nonfasting “OR (“Postcibal Period”) AND (“Normal population”) OR (“Healthy volunteers”) OR (“Healthy subject”). The search technique used text words and controlled phrases for the normal population’s fasting and nonfasting lipid profiles. The studies were included according to the preferred reporting items for systematic reviews and meta-analyses. (See Appendix 1).

### Study selection

#### Inclusion criteria

Studies satisfying the following criteria were included:

- Study design: All clinical trials or observational studies that measured lipid profiles in fasting and postprandial states.
- Population: A population of individuals aged between 18 and 75 years who are in good health. Establishing a baseline by accounting for the influence of various diseases eliminated any potential variables that could impact the results of lipid profile testing. Therefore, the specific effects of diet on the lipid profile were isolated and analyzed.
- Outcome: Studies reporting demographic and laboratory findings.
- Language: Only studies published in international scientific journals and written in English were included.
- Studies that had enough information for qualitative and quantitative analyses.

#### Exclusion criteria

- The researchers did not suggest sufficient data.
- Assessing lipid profile parameters or comparing the concentrations of different lipid parameters in unhealthy individuals were omitted.
- Animal research, posters, duplicate papers, or conference papers were not included.

### Screening and study selection

**Fig. 2:**
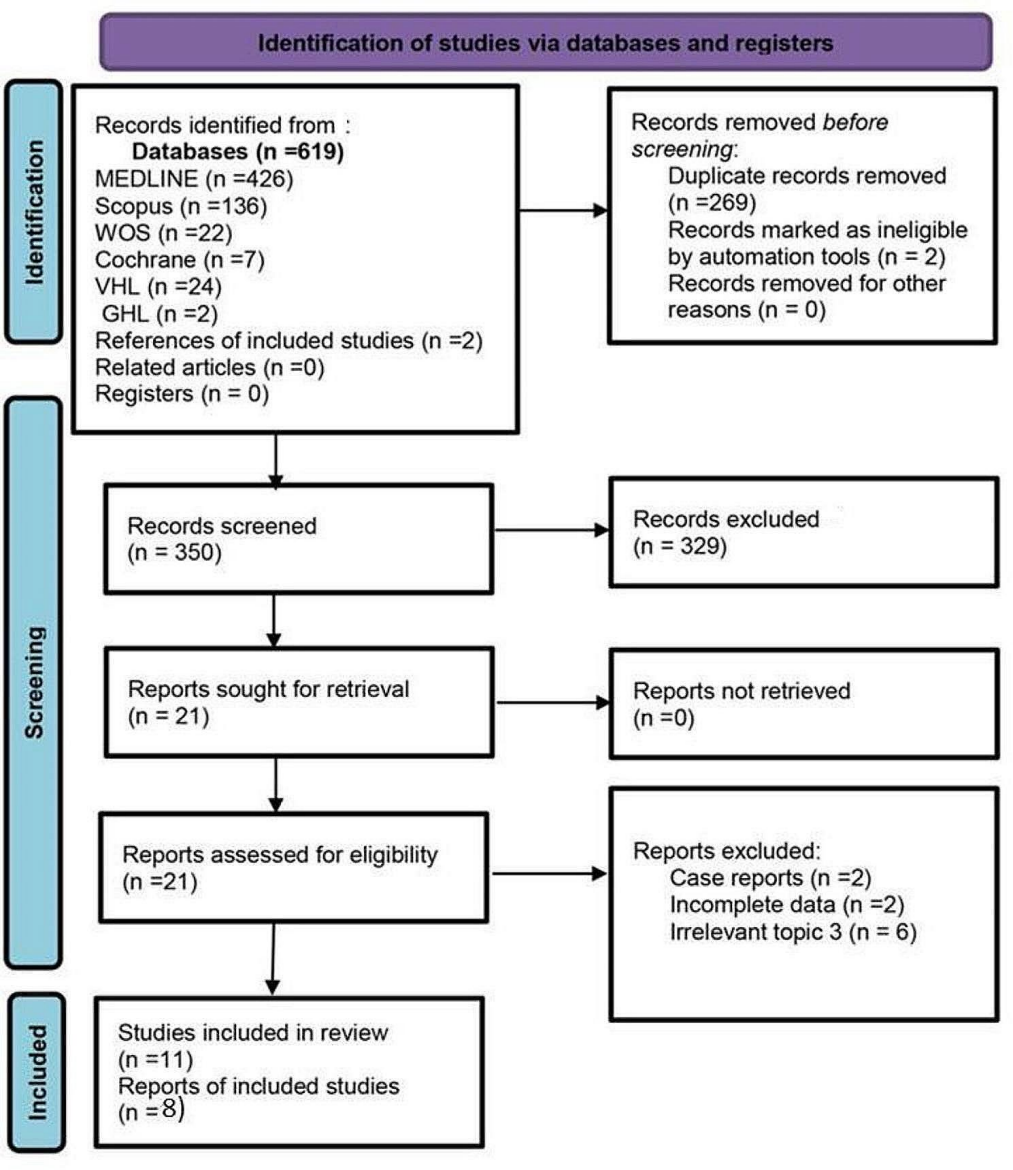
Identification of studies via databases and registers (Lipid Testing)

The studies were exported to EndNote X9.1 (Clarivate Analytics, https://clarivate.com/) to remove duplicates. Two independent reviewers [HS, AB] screened all records for eligibility. Eligibility screening was performed in two steps: in the first step, titles and abstracts were screened, and in the second step, full-text articles of the selected abstracts were retrieved and assessed for eligibility. Disagreements were resolved by discussion with a third reviewer. The following PRISMA diagram illustrates the search procedure and details of the study selection process in Fig. 2.

### Data extraction

Data about the patients’ demographic features, past medical history, clinical presentation, laboratory values, therapies, and clinical outcomes were extracted. Two reviewers, working independently, collected the data from a standardized Microsoft Excel spreadsheet. To ensure the accuracy of the retrieved data, an additional reviewer, independent from the previous two, conducted a thorough examination. All instances of disputes were effectively resolved by engaging in thoughtful and constructive debates.

### Evaluation of the bias risk of the included studies

The quality of the included studies was assessed using the National Institute of Health (NIH) scale for observational studies.

#### Assessing risk of bias in individual studies

Two authors (AB and HS) evaluated the reliability of the studies using the National Institutes of Health (NIH) quality assessment tool for various types of research, including observational cohort, case‒control, controlled interventional, and cross-sectional studies [20]. This instrument comprises a set of 14 inquiries of various aspects, such as sample size, selection process, exposure assessment, and outcome evaluation. Research articles with a score of 9 or more points were classified as having good quality, while those scoring between 5 and 8 points were deemed to have reasonable quality. Articles with scores ranging from 1 to 4 were categorized as having low quality.

#### Assessing the risk of bias across studies

The results from all the studies were thoroughly scrutinized and compared to assess any potential bias in the evaluated trials. This enabled the researchers to detect and eliminate biased reporting of outcomes. Egger and colleagues found that the reliability of detecting publication bias using the funnel plot method fails when there are fewer than ten pooled studies [21].

### Data synthesis and analysis

Review Manager Software Version 5.3 (Rev-Man 5.3, Copenhagen, The Nordic Cochrane Centre, The Cochrane Collaboration, 2020). Four studies reported the mean and standard deviation [5, 11, 13, 14]. Another four studies reported the median and range [15, 23–25]. For the statistical analysis, the data are presented as the means and standard deviations, so the data were transformed into means and standard deviations according to the methods described by McGrath [22].

### Heterogeneity

The evaluation of heterogeneity involved a visual examination of the forest plots to verify the extent of overlap between the 95% confidence intervals of the pooled estimations. The chi-square test was employed to assess heterogeneity, while the I2 test was used to quantify heterogeneity. The heterogeneity of the outcomes was deemed significant when the *P* value exceeded 0.1 and 2 was > 50%. Evidence of heterogeneity in the LDL-cholesterol and triglyceride data was observed in the present study. A random-effects model was employed to address this heterogeneity. Additionally, sensitivity analysis, subgrouping analysis, and prediction intervals were calculated to assess the impact of heterogeneity on the study outcomes and determine its magnitude (trivial, moderate, or substantial).

*P* values less than 0.05 for the overall standardized mean difference (SMD) were considered to indicate statistical significance. UN inconsistency (I2), chi-square (X2), and tau-square tests were used to assess heterogeneity.

#### Sensitivity analysis

To evaluate the influence of each study on the overall results, a leave-one-out analysis was conducted to address the variability observed in LDL cholesterol levels. In addition, a specific subgroup analysis was performed for TG. A study that significantly deviated from the norm was excluded to assess the collective effect and accommodate potential variations.

#### Subgrouping analysis

Subgrouping analysis was conducted based on patients’ metabolic status by separating countries into fat-rich and fat-poor meal countries.

#### Calculation of the 95% prediction interval

The summary meta-analysis estimates M, the two-sided crucial t value t1-0.05/2, k-1, and the standard deviation for the prediction interval (SDPI) are required to construct the 95% prediction interval. With k being the number of papers included in the meta-analysis, DF = k-1 and a probability level of 0.025 are used. The SDPI, also known as the standard deviation of the prediction interval, has the formula SDPI = (τ2 + SE2), where τ2 is the estimated heterogeneity and SE denotes the standard error of the SMD. If the SE was not supplied, its estimated value could be calculated by multiplying the separation between the 95% confidence interval for the SMD by 3.92. The 95% confidence intervals of the bottom and upper boundaries are equal to M t1-0.05/2 and k-1 SDPI, respectively.

## Results

### Details of the included studies

Eight studies were included, with 244,665 participants matched by age and sex. Seven studies (Cartier et al., 2017 [5]; Sidhu and Naugler, 2012 [11]; Yanget al., 2018 [13]; Langston, 2008 [15]; and Umakanth and Ibrahim, 2018 [24]; Liu et al., 2021 [25]; Szternel et al., 2019 [23]) reported separate measurements of lipid parameters in fasting and usual diet lifestyles. Schaefer et al., 2001 [14] reported separate measurements of lipid parameters during fasting and after four hours of a fat-rich meal. All studies that reported different fasting and nonfasting lipid parameter values were included in the meta-analyses for comparison (Table 1).

**Table 1:** Features of the included studies

Study	Ref.	Country	Method	Assay	Kits	Device	Main Outcome (for each study)
Cartier et al., (2017)	[ [5]]	Canada	Cohort	ECM	Roche diagnostics	Abbott chemistry analyzer (architect c 16,000)	Differences reported in TG &LDL-Chol.
Sidhu and Naugler (2012)	[ [11]]	Canada	CS	ECM	Roche diagnostics	Modular analyzer	differences reported in all parameters
Yang et al. (2018)	[ [13]]	China	Cohort	ECM	Roche diagnostics	Dilution mass spectrometry	Differences reported in LDL-Chol.
Schaefer et al., (2001)	[ [14]]	USA	RCT	2-RE, CASC	Genzyme Diagnostics Cambridge Massachusetts	Abbott Spectrum CCx analyzer (Abbott-diagnostics, Irving, Texas)	Differences reported in TG &LDL-Chol.
Langsted (2008)	[ [15]]	Denmark	CS	Nr	Nr	Nr	differences reported in all parameters
Umakanth and Ibrahim (2018)	[ [24]]	Srilanka	Cohort	Nr	Nr	Nr	Differences reported in TG &LDL-Chol.
Liu (2021)	[ [25]]	China	CS	ECM	BioSino Biotechnology Kit & Science Inc., Beijing, China	Hitachi 7150, Tokyo, Japan	differences reported in all parameters except HDL-chol
Szternel (2019)	[ [23]]	Poland	Cs	ECM	Randox Laboratories (Crumlin, UK)	Horiba ABX Pentra 400 analyzer (Horiba ABX, Montpellier, France)	Differences reported in TG only

CS, Cross-section; RCT, randomized clinical trial; ECM, enzymatic colorimetric method; 2-RE, 2-reagent enzymatic; CASC, colorimetric assays having a sensitive chromosphere; Nr, not reported.

### Characteristics of the included studies

Table 2 was constructed to present the data extraction. Four cross-sectional studies were identified: Sidhu & Naugler, 2012 [11]; Langsted et al., 2008 [15]; Liu et al., 2021 [25]; Szternel et al., 2019 [23]; the first study [11] involved 209,180 subjects representing 46.9% males and 53.1% females with a mean age of 52.8 years (18–74 years) and no available data for those participants; the second study [15] enrolled 33,391 subjects representing 47% males and 53% females with a mean age of 60 ± 9.5 years and a BMI of 26.5 ± 2.5; the third study [25] enrolled 499 participants divided into 51.6% males and 49.4% females with a mean age of 55 ± 13 years; and the fourth study [23] involved 289 participants distributed into 50.9 males and 49.1 females with a median age of 48 ± 1.36 years. Additionally, three cohort studies were detected: Cartier et al., 2017 [5]. In this study, individuals with diabetes were compared to a control group. The control arm was chosen for examination and included 1093 subjects, 50.3% male and 42.5% female, with a mean age of 62.5 ± 10 years. The study conducted by Yang et al., 2018 [13] involved 41,55% male and 45% female participants, with a mean age of 25.6 ± 6.2 years and a BMI of 21.6 ± 6.2 years. Umakathand Ibrahim 2018 [24] included 84 participants; 64.28% were male, and 35.71% were female aged 25 to 60. Finally, the RCT by Schaefer et al. 2001 [14] (this study compares CVs to controls; only the control group was chosen for the study) included 88 subjects, 85% male and 15% female, with a mean age of 62 ± 8.6 years and BMI of 26.2 ± 4.2 years.

**Table 2:** Characteristics of the included participants

Study	Number of part.	% Males	% Females	Age M ± SD/M (range)/Range)	Height	Weight	BMI
Cartier et al., (2017)	1093	50.3	42.5	62.6 ± 10	Nr	Nr	Nr
Sidhu and Naugler (2012)	209,180	46.9	53.1	52.8 (18–74)	Nr	Nr	Nr
Yang et al., (2018)	41	55	45	25.65 ± 6.2	165.8 ± 57	60 ± 17.4	21.65 ± 2.6
Schaefer et al., (2001)	88	85	15	62 ± 8.6	173.9 ± 9.1	79.4 ± 15.2	26.2 ± 4.2
Langsted (2008)	33,391	47	53	60 ± 9.5	Nr	Nr	26 ± 2.5
Umakanth and Ibrahim (2018)	84	64.28	35.71	25–60	Nr	Nr	Nr
Liu (2021)	499	51.6	48.4	55 ± 13	Nr	Nr	24.6 ± 3.7
Szternel (2019)	289	47.4	52.6	48 ± 1.36	143 ± 7.5	36.5 ± 8	17.8

Nr, not reported.

### Quality assessment

The quality of the included studies was assessed using the NIH scale. Six studies scored 9, 10, 11, 11, 12, and 10; Schaefer et al., 2001 [14], Langsted, 2008 [15], Yang et al., 2018 [13], Sidhu and Naugler 2012 [11], Liu et al., 2021 [25] and Szternel et al., 2019 [23], respectively, and were considered high-quality, while two studies, Cartier et al., 2017 [5] and Umakanth and Ibrahim, 2018 [24], were targeted (score 8) with fair quality (Table 3).

**Table 3:** Quality assessment of the included studies according to the National Institute of Health (NIH) quality assessment tool for observational cohort and cross-sectional studies

Study	C1	C2	C3	C4	C5	C6	C7	C8	C9	C10	C11	C12	C13	C14	Score
Cartier et al., (2017) [ [5]]	Yes	Yes	Yes	No	Yes	Yes	No	No	Yes	No	Yes	No	Yes	CD	8
Sidhu and Naugler (2012) [ [11]]	Yes	Yes	Yes	No	Yes	Yes	Yes	Yes	Yes	Yes	Yes	No	Yes	CD	11
Yang et al., (2018) [ [13]]	Yes	Yes	Yes	Yes	Yes	Yes	No	yes	Yes	Yes	Yes	No	Yes	CD	11
Schaefer et al., (2001) [ [14]]	Yes	Yes	Yes	No	Yes	Yes	Yes	No	Yes	No	Yes	No	Yes	CD	9
Langsted (2008) [ [15]]	Yes	Yes	Yes	No	Yes	Yes	Yes	Yes	Yes	No	Yes	No	Yes	CD	10
Umakanth and Ibrahim (2018) [ [24]]	Yes	No	Yes	Yes	Yes	Yes	Yes	No	Yes	No	No	No	Yes	CD	8
Liu (2021) [ [25]]	Yes	Yes	Yes	Yes	Yes	Yes	Yes	Yes	Yes	Yes	Yes	No	Yes	CD	12
Szternel (2019) [ [23]]	Yes	Yes	Yes	No	Yes	Yes	Yes	No	Yes	Yes	Yes	No	Yes	CD	10

C, criterion; CD, cannot be determined; NA, not applicable; Criterion 1, clear statement of research question and objective; Criterion 2, clear specification and definition of the study population; Criterion 3, participation rate of 50% of eligible persons; Criterion 4, selection of subjects from the same population and time period and application of selection criteria on all subjects uniformly; Criterion 5, justification of sample size; Criterion 6, measurement of exposure before measurement of outcome; Criterion 7, sufficient timeframe to predict an association between exposure and outcome; Criterion 8, examination of different levels of the exposure in relation to the outcome; Criterion 9, clear and valid definition of exposure measures and its consistent implementation on study subjects; Criterion 10, assessment of exposure more than once over time; Criterion 11, clear and valid definition of outcome measures and its consistent implementation on study subjects; Criterion 12, blinding of outcome assessors to exposure status of participants; Criterion 13, loss to follow up being 20% or less; Criterion 14, measurement or statistical adjustment of confounding variables.

**Fig. 3:**
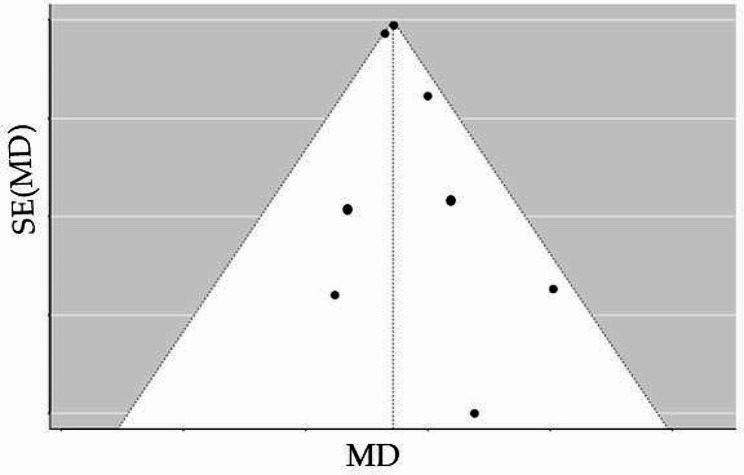
Funnel plot for publication bias

A funnel plot is not accurate for the assessment of publication bias in this study (fewer than ten studies), so Egger’s regression was utilized, revealing significance for publication bias (*P* < 0.001). Subsequently, publication bias was assessed using Egger’s equation. Based on the refilled and trimmed number of studies in Table 4, a renewed search across databases was conducted to identify an additional two studies—Liu (2021) [25] and Szternel (2019) [23]—to conceal publication bias across the studies (Fig. 3; Table 4).

**Table 4:** Eggers regression for publication bias

Publication Bias Assessment					
**Test Name**		**Value**		***P***	
Fail-Safe N		250.000		< 0.001	
Begg and Mazumdar Rank Correlation		− 0.067		1.000	
Egger’s Regression		0.966		0.334	
Trim and Fill Number of Studies		2.000		.	

Note. Fail-safe N Calculation Using the Rosenthal Approach

### Differences in fasting and nonfasting cholesterol and high-density cholesterol

**Fig. 4:**
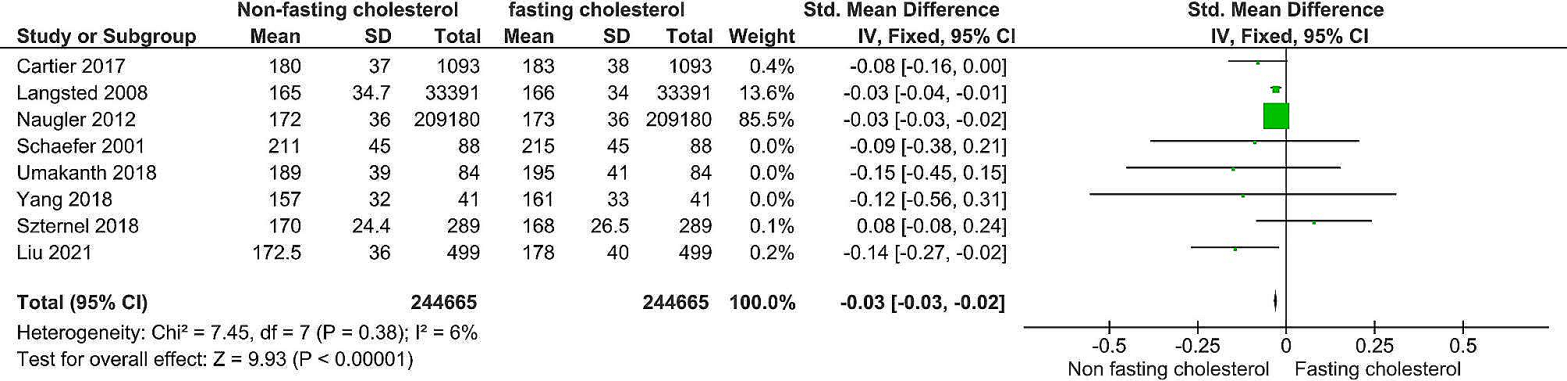
Forest plot of cholesterol

**Fig. 5:**
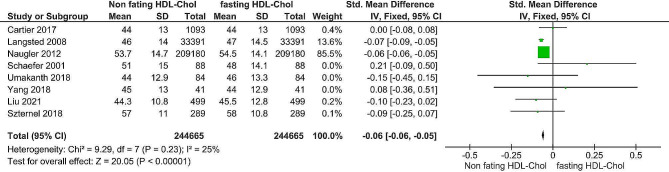
Forest plot of HDL- cholesterol

As depicted in Figs. 4 and 5, the estimated mean differences in cholesterol and high-density lipoprotein levels between fasting and nonfasting patients were − 0.03 − 0.02 and − 0.06 − 0.05, respectively. The overall impact of both metrics was significant (*P <* 0.00001). The Z values were 9.93 and 20.05 for cholesterol and high-density lipoprotein, respectively. The X2 values were 7.45 (*P* = 0.38) and 9.29 (*p* = 0.23) for testing heterogeneity, respectively. The I2 statistics for cholesterol levels, fasting and nonfasting lipoprotein levels, and high-density lipoprotein levels were I2 = 6 and I2 = 25%, respectively; therefore, a fixed-effects model was employed due to the homogeneity observed in the included studies.

### Fasting and nonfasting triglyceride levels and low-density cholesterol differences

**Fig. 6:**
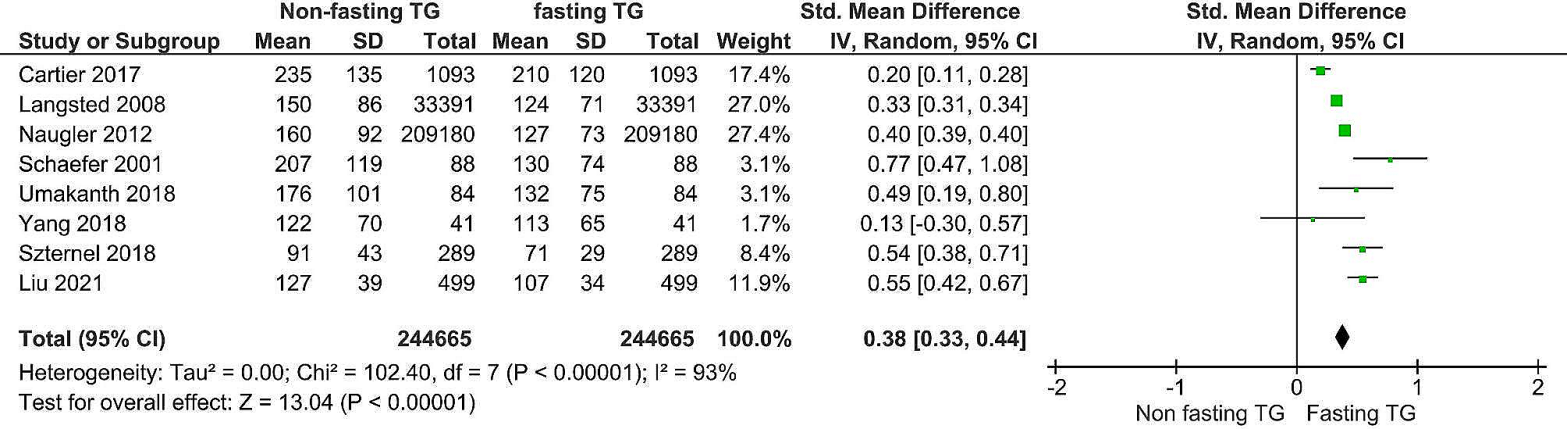
Forest plot of triglycerides

**Fig. 7:**
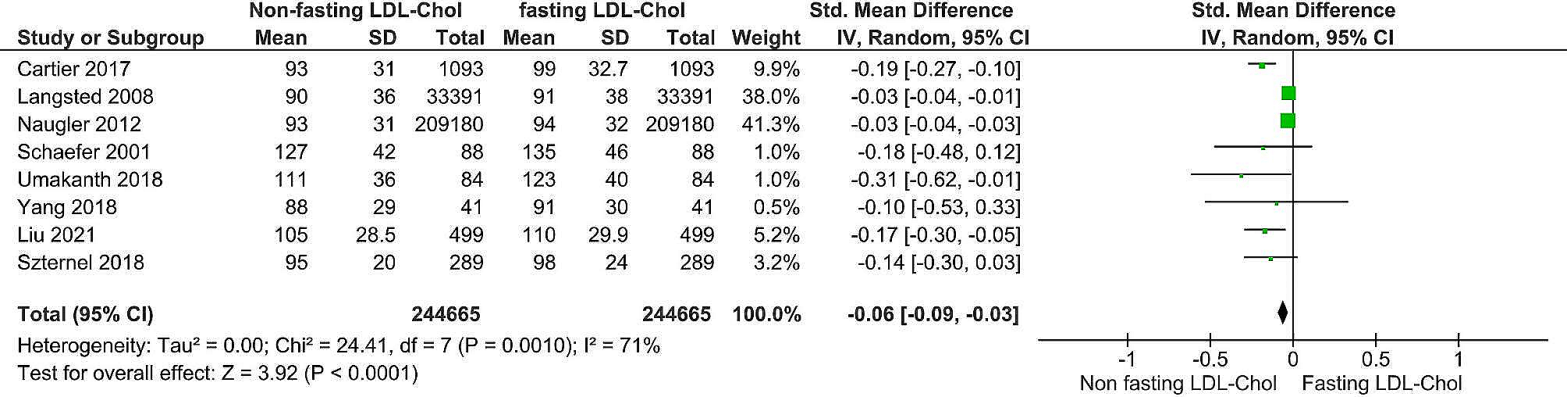
Forest plot of LDL- cholesterol

As shown in Figs. 6 and 7, the estimation mean differences in triglycerides and low-density lipoprotein levels between fasting and nonfasting patients were 0.38 (95% CI, 0.44) and − 0.06 (95% CI, -0.09), respectively. For both metrics, the test for the total effect was significant (*P* < 0. 00001), and the Z values were 13.04 and 3.92 for triglycerides and low-density lipoproteins, respectively. For testing heterogeneity, the X2 values were 102.4 (*P* < 0.00001) and 24.4 (*P* = 0.001). The I2 statistics for TG levels, fasting and nonfasting lipoprotein levels, and low-density lipoprotein levels were I2 = 93 and I2 = 71%, respectively. A random-effects model was utilized due to the significant heterogeneity observed in the included studies. Sensitivity and subgrouping analyses were conducted, and the prediction intervals were discussed.

### Sensitivity analysis for LDL-chol

A random-effects model was employed due to significant heterogeneity in the included studies, and a sensitivity analysis for LDL-C was also conducted. Leaving out Cartier, 2017 [5] resolved the heterogeneity in Appendix 2.

By excluding one study from each scenario, heterogeneity was not resolved, so the subgrouping analysis was conducted based on patients’ metabolic status by separating countries into fat-rich meal and fat-poor meal countries (Appendix 3). The subgroup analysis resolved heterogeneity (X2 = 0.57, *P* = 0.45, I2 = 0%). Additionally, prediction intervals were discussed.

## Discussion

The characteristics of the included studies, including the study design, participant demographics, and quality assessment scores, were detailed. Most of the studies were of high quality, as indicated by their NIH scores. However, two studies were rated as being of fair quality, emphasizing the need to interpret their results carefully.

The analysis revealed significant differences in cholesterol and high-density lipoprotein levels between fasting and nonfasting states, as evidenced by estimated mean differences and corresponding confidence intervals. Heterogeneity testing and model selection were conducted based on the I2 statistics, with a fixed-effect model utilized for homogenous data and a random-effect model for heterogeneous data.

Regarding cholesterol, a significant difference between fasting and nonfasting levels could be seen in the forest plot (Fig. 4). The overall SMD was − 0.03, and the 95% confidence interval (CI) was (-0.03, -0.02), with a *P* value < 0.00001. Regarding heterogeneity, I2 = 6%, and I2 is the percentage of observed variance that reflects actual effect size variations instead of sampling error. The findings align with studies with larger sample sizes: Sidhu and Naugler., 2012 [11]; Langsted., 2008 [15] and Liu et al., 2021 [25]. A large sample size is crucial for minimizing the standard deviation around the mean and, as a result, reducing error. These findings align with previous studies showing the superiority of larger sample sizes over smaller ones. These studies include Cartier et al., 2017 [5], Yang et al., 2018 [13], Schaefer et al., 2001 [14], Umakanth and Ibrahim., 2018 [24] and Szternel et al., 2019 [23].

In addition, the forest plot revealed a notable disparity in HDL levels between individuals who fasted and those who did not. The overall standardized mean difference is -0.06, with a 95% confidence interval of (-0.06, -0.05) and a *P* value of less than 0.00001. Regarding heterogeneity, an I2 value of 42% and a *P* value of less than 0.12 suggested that a relatively small proportion of the overall observed effect size variance was true. This study aligns with the findings of several previous researchers, such as Sidhu and Naugler, 2012 [11]; Langsted., 2008 [15]; Liu et al., 2021 [25]; and Szternel et al., 2019 [23], and disagrees with Cartier et al., 2017 [5]; Yang et al., 2018 [13]; Schaefer et al., 2001 [14]; and Umakanth and Ibrahim., 2018 [24].

The forest plot also revealed a statistically significant difference in triglyceride levels between fasting and nonfasting patients. The overall SMD was 0.38, the 95% CI was 0.33, 0.44, and the Z value of the overall effect was 13.04, with a P value < 0.00001. That is, fasting was significantly different from nonfasting. According to the prediction intervals, triglyceride levels ranged from 0.25 to 1.21; this study expected most levels (moderate effect) to coincide with the respective CIs of overall effect (0.28, 0.41), trivial levels with a range of 0.25 to 0.28 and substantial accurate effect levels with a range of 0.41 to 1.21. Both the PI and overall CI of triglycerides were on the same positive side as the null, i.e., fasting was significantly different from nonfasting in the present study and future studies. All studies’ point estimates and 95% CIs were in the positive direction of the null line, except for Yang et al., 2018 [13]. In the Schaefer et al., 2001 [15] study, the SMD was within the overall range, but few values within the 95% confidence intervals (CIs) indicated a substantial actual effect of the PI. According to Cartier et al., 2017 [5], the SMD and its 95% CI had a trivial effect on the PI. In Langsted, 2008 [15], Umakanth and Ibrahim 2018 [24], Liu et al., 2021 [25] and Szternel et al., 2019 [23], the SMD and its 95% CI were found to have substantial effects on the PI. In Yang et al. [13]. , although the 95% CI crossed the null line in the negative direction, its point estimate value was within the trivial effect of the PI.

According to the LDL data analysis, the forest plot showed a significant difference between fasting and nonfasting levels. The overall standardized mean difference was − 0.06 (95% CI (-0.09, -0.03)), and the Z value of the overall effect was 3.92 (*P* < 0.0001); i.e., nonfasting significantly differed from fasting (*P* < 0.05). In the studies of Cartier et al., 2017 [5], Sidhu and Naugler, 2012 [11], Yang et al., 2018 [13], Schaefer et al., 2001 [14], Langsted, 2008 [15], Umakanth and Ibrahim, 2018 [24] and Liu et al., 2021 [25], the SMD had a negative effect on the null line, with only 95% CI of Yang et al., 2018 [13], Schaefer et al., 2001 [14] and Szternel et al., 2019 [23], which were in the positive direction of the null line, i.e., a substantial effect of the PI. In the studies of Yang et al., 2018 [13], Sidhu and Naugler., 2012 [11], and Langsted, 2008 [15], the SMD and 95% CI were within the overall moderate effect of the PI. However, Umakanth and Ibrahim, 2018 [24] showed that the SMD and 95% CI were within the trivial range of the effect of the PI.

Similarly, differences in triglyceride and low-density lipoprotein levels between fasting and nonfasting states were observed, with significant effects demonstrated through estimated mean differences and heterogeneity testing. A random-effects model was employed due to significant heterogeneity among the included studies, necessitating sensitivity and subgrouping analyses to explore potential sources of variation.

Hence, most of the included studies used Friedewald’s equation; logically, TG levels in blood were inversely proportional to LDL-cholesterol levels, and normal levels of serum TG and LDL-cholesterol ranged from 150 to 200 mg/dL and < 135 mg/dL, respectively, because TG, which represents 25%, is not a significant component of LDL-chol, but cholesterol, which represents 75% of LDL-chol. In the fasting state, TG is used for energy production so that the levels of total TG decrease and LDL cholesterol increase. This explains why total TG is on the positive side and LDL-C is on the negative side.

The previous results for all lipid profiles matched and explained according to Kovar and Havel, 2002 [26], Nakajima et al., 2011 [27], and Feingold, 2021 [28], who stated that the appearance of chylomicrons in the blood is followed by a rise in very low-density lipoproteins (VLDLs) due to competition for lipolysis between VLDL and chylomicrons [26, 27]. Postprandial lipaemia results from an increase in both intestine-derived chylomicrons and liver-derived VLDL [29]. Capillary endothelial cells have an enzyme called lipoprotein lipase (LPL) on their luminal surface, which binds to chylomicrons and hydrolyses their triglycerides, releasing free fatty acids (FFAs) that may easily pass into cells and be oxidized for energy or re-esterified for cholesterol ester enrichment [30]. ApoB48 and ApoE levels are preserved throughout the conversion of chylomicrons to chylomicron remnants. The liver is the primary organ that removes remnants from the blood; receptors for chylomicron remnants recognize ApoE and take up the remnants. Therefore, postprandially, the amount of VLDL tends to increase more than that of chylomicrons [27, 31]. After six hours, VLDL is converted to LDL in circulation. Peristalsis helps pump chyme into the small intestine while you eat. They occur during digestion and can persist for two hours after the stomach is emptied. It takes four to five hours for the stomach to empty into the small intestine after a meal [27, 32].

The American Heart Association (AHA) guidelines do not recommend a fasting protocol for estimating the risk of atherosclerotic cardiovascular disease. However, the AHA only supposes fasting lipid testing for patients who will undergo statin therapy as well as for patients in whom the non-HDL cholesterol level is less than 5.7 mmol/L (220 mg/dL) or triglycerides are greater than 5.7 mmol/L (500 mg/dL) to avoid the effect of lipemic serum. Nonfasting and fasting results should be complementary but not exclusive because these could be signs of hereditary and/or secondary causes of hypertriglyceridemia [7, 33]. According to the findings of Wilson et al. [34], the identification of potentially actionable abnormal lipid test results, explicitly fasting triglyceride (TG) levels equal to or exceeding 500 mg/dL, necessitates the reporting of such cases as hypertriglyceridemia. Enhancing the proper utilization and accurate documentation of lipid tests is expected to improve their efficacy in the comprehensive care of individuals with a heightened susceptibility to atherosclerotic cardiovascular disease (ASCVD) occurrence. On a laboratory basis, if lipemic serum is detected, fasting for 8–12 h for triglyceride and LDL testing is mandatory; in addition, LDL should be technically measured using diagnostic kits, not Friedewald’s formula. This is because lipaemia affects the calculation of LDL cholesterol, and chylomicrons affect the measurement of triglycerides.

Specifically, sensitivity analysis for low-density lipoprotein cholesterol was performed, and individual studies were excluded to assess their impact on heterogeneity. Subgrouping analysis based on patients’ metabolic status and dietary habits was also conducted to explore sources of heterogeneity further and refine the study’s findings. According to the current statistical data, most lipid measurements, including cholesterol, HDL cholesterol, lipoprotein triglycerides, and LDL, showed significant changes between fasting and nonfasting testing protocols [35]. .

## Strengths and limitations of the study

First, this study identified eight studies involving a large sample size of 244,665 participants, matched by age and sex, and reported separate measurements of lipid parameters under fasting and nonfasting conditions; these studies allowed for a comprehensive analysis of the differences in lipid profiles between fasting and nonfasting states. Second, it is important to note that the smaller trials did not show any variation between fasting and nonfasting patients. However, a larger study with a larger sample size revealed a significant difference, which aligns with the study’s findings. This study has two limitations: a restricted number of included studies due to stringent inclusion and exclusion criteria and significant heterogeneity observed among studies regarding triglycerides and LDL cholesterol.

## Conclusion

A meta-analysis of lipid profiles revealed significant differences between fasting and nonfasting states, emphasizing the importance of fasting for consistent results. Fasting status strongly influences cholesterol, HDL, triglyceride, and LDL levels, which are crucial for cardiovascular risk assessment. Clinicians must consider fasting status when interpreting lipid tests, especially in metabolic conditions such as diabetes, to guide therapy effectively. This study underscores the need for fasting-specific lipid testing guidelines for personalized cholesterol therapy and improved cardiovascular risk management.

### Electronic supplementary material

Below is the link to the electronic supplementary material.

Supplementary Material 1

Supplementary Material 2

Supplementary Material 3

Supplementary Material 4

Supplementary Material 5

Supplementary Material 6

Supplementary Material 7

## Data Availability

The data in the current paper are publicly available since this is a meta-analysis conducted based on the cited literature.

## Declarations

### Systematic review registration

PROSPERO CRD42022376871.

## Abbreviations

Chol: Cholesterol
CI: Confidence interval
HDL-Chol: High-density lipoprotein cholesterol
I2: Inconsistency
LDL-Chol: Low-density lipoprotein cholesterol
SMD: Standard mean difference
TG: Triglycerides
X2: Chi-square test
