# Supplementary material for: Unraveling the controversy between fasting and nonfasting lipid testing in a normal population: a systematic review and meta-analysis of 244,665 participants
Source: Lipids Health Dis. 2024 Jun 27;23:199. doi: 10.1186/s12944-024-02169-y (PMC11210154; doi:10.1186/s12944-024-02169-y)
Supplement: Supplementary file 1 — Supplementary Material 1 [file 12944_2024_2169_MOESM1_ESM.docx]

**PubMed Search**

**1-((("Fasting"[Mesh]) AND "Postprandial Period"[Mesh]) AND "Lipids"[Mesh]) AND "Healthy Volunteers"[Mesh] ………38 Results**

**2-(((Fast*[Title/Abstract]) AND ((Lipids[Title/Abstract]) OR (Ceroids[Title/Abstract] OR Fats[Title/Abstract] OR Fatty acids[Title/Abstract] OR Glycerides[Title/Abstract] OR Glycolipids[Title/Abstract] OR Lipoproteins[Title/Abstract] OR Lipopolysaccharides[Title/Abstract]))) AND ((Normal population[Title/Abstract]) OR (Healthy volunteers[Title/Abstract]))) AND (((Nonfast[Title/Abstract]) OR (Non-fast*[Title/Abstract])) OR (Postprandial[Title/Abstract]))……………..59Results**

**3-((Intermittent Fasting OR Hunger Strike OR Time Restricted Feeding OR fast*) AND (Postprandial Periods OR Postcibal Periods OR nonfast* OR non-fast* ) AND (Lipids OR Ceroids OR Fats OR Fatty acids OR Glycerides OR Glycolipids OR Lipoproteins OR Lipopolysaccharides) AND (Normal population OR Healthy volunteers OR Healthy subject))" …….. (329 Results )**

**Cochrane Search**

**((Intermittent Fasting OR Hunger Strike OR Time Restricted Feeding OR fast*) AND (Postprandial Periods OR Postcibal Periods OR nonfast* OR non-fast* ) AND (Lipids OR Ceroids OR Fats OR Fatty acids OR Glycerides OR Glycolipids OR Lipoproteins OR Lipopolysaccharides) AND (Normal population OR Healthy volunteers OR Healthy subject))" ( 7Results)**

**WOS Search**

**lipid*** (Title) or **ceroids** (Title) or **fats** (Title) or **fattyacid** (Title) or **glycerides** (Title) or **glycolipids** (Title) or **lipoproteins** (Title) or **lipopolysaccharides** (Title) AND **fast*** (Title) or **Intermittent Fasting** (Title) or **Hunger Strike** (Title) or **Time Restricted Feeding** (Title) AND **postprandial** (Title) or **non fast*** (Title) or **postcibal** (Title) AND **normal population** (Title) or **Healthy volunteer** (Title) or **Healthy subjects** (Title) …….(22)

**Scopus Search**

( ( TITLE-ABS-KEY ( time AND restricted AND feeding ) OR TITLE-ABS-KEY ( intermittent AND fasting ) OR TITLE-ABS-KEY ( hunger AND strike ) OR TITLE-ABS-KEY ( fast* ) ) ) AND ( ( TITLE-ABS-KEY ( postprandial AND periods ) OR TITLE-ABS-KEY ( postcibal AND periods ) OR TITLE-ABS-KEY ( nonfast* ) OR TITLE-ABS-KEY ( non-fast* ) ) ) AND ( ( TITLE-ABS-KEY ( lipids ) OR TITLE-ABS-KEY ( ceroids ) OR TITLE-ABS-KEY ( fats ) OR TITLE-ABS-KEY ( fatty AND acids ) OR TITLE-ABS-KEY ( glycerides ) OR TITLE-ABS-KEY ( glycolipids ) OR TITLE-ABS-KEY ( lipoproteins ) OR TITLE-ABS-KEY ( lipopolysaccharides ) ) ) AND ( ( TITLE-ABS-KEY ( normal AND population ) OR TITLE-ABS-KEY ( healthy AND volunteers ) OR TITLE-ABS-KEY ( healthy AND subject ) ) )….(136)Result

VHL Regional Portal

(Intermittent Fasting OR Hunger Strike OR Time Restricted Feeding OR fast*) AND (Postprandial Periods OR Postcibal Periods OR nonfast* OR non-fast* ) AND (Lipids OR Ceroids OR Fats OR Fatty acids OR Glycerides OR Glycolipids OR Lipoproteins OR Lipopolysaccharides) AND (Normal population OR Healthy volunteers OR Healthy subject)…..(24 Results)

Global Index Medicus

(tw:(Intermittent Fasting OR Hunger Strike OR Time Restricted Feeding OR fast*)) AND (tw:(Postprandial Periods OR Postcibal Periods OR nonfast* OR non-fast* )) AND (tw:(Lipids OR Ceroids OR Fats OR Fatty acids OR Glycerides OR Glycolipids OR Lipoproteins OR Lipopolysaccharides)) AND (tw:(Normal population OR Healthy volunteers OR Healthy subject))…..2 results
