# Supplementary material for: Unraveling the controversy between fasting and nonfasting lipid testing in a normal population: a systematic review and meta-analysis of 244,665 participants
Source: Lipids Health Dis. 2024 Jun 27;23:199. doi: 10.1186/s12944-024-02169-y (PMC11210154; doi:10.1186/s12944-024-02169-y)
Supplement: Supplementary file 3 — Supplementary Material 3 [file 12944_2024_2169_MOESM3_ESM.pdf]

Academic Perfection

Certificate of Editing

This document serves to confirm that the manuscript listed below has undergone professional English language editing to achieve academic excellence. To authenticate this certification, you scan the QR code provided below.

Manuscript Title

Unravelling The Controversy Between Fasting And Non-Fasting Lipid Testing In Normal Population: A Systematic Review And Meta-Analysis For 244.665 Participants

Authors

Ahmed B. Zaid, Samah M. Awad, Mona G. Elabd, Sara A. Said, Shimaa K. Almahy, AbdulRahman A Saied, Alshimaa M. Elmalawany, Hind S. Aboshabaan, Helmy S. Saleh

Date Issued

April 19th, 2024

Issued by

Michael Abdelmasseh

Academic Perfection

Michael Abdelmasseh MD MPH

General Manager

Email: academicperfection1@gmail.com

Certificate No: 16048
