# Supplementary figures and images for: Unraveling the controversy between fasting and nonfasting lipid testing in a normal population: a systematic review and meta-analysis of 244,665 participants
Source: Lipids Health Dis. 2024 Jun 27;23:199. doi: 10.1186/s12944-024-02169-y (PMC11210154; doi:10.1186/s12944-024-02169-y)

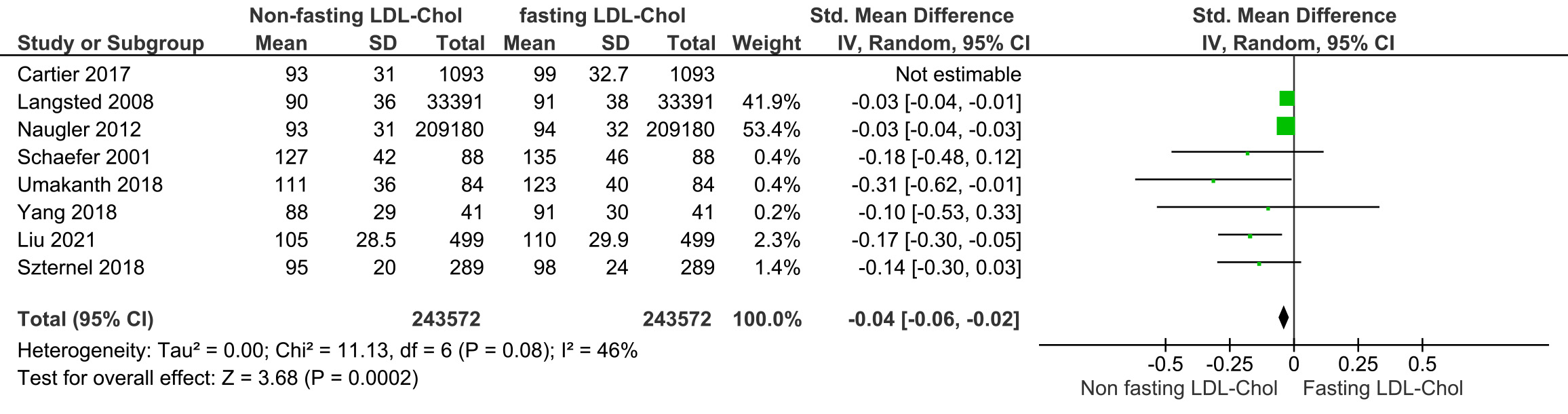

Supplement: Supplementary file 4 — Supplementary Material 4 [file 12944_2024_2169_MOESM4_ESM.jpg]

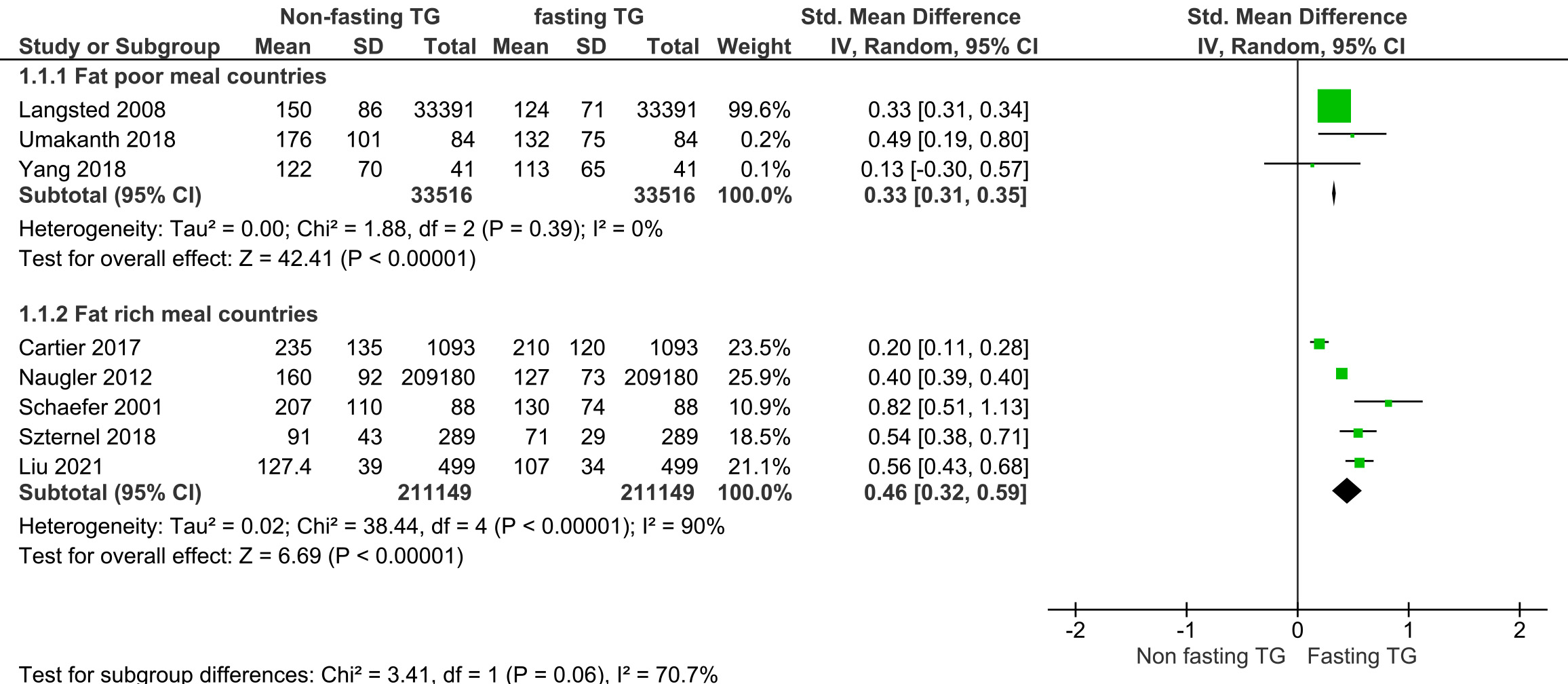

Supplement: Supplementary file 6 — Supplementary Material 6 [file 12944_2024_2169_MOESM6_ESM.jpg]
