# Supplementary material for: Unraveling the controversy between fasting and nonfasting lipid testing in a normal population: a systematic review and meta-analysis of 244,665 participants
Source: Lipids Health Dis. 2024 Jun 27;23:199. doi: 10.1186/s12944-024-02169-y (PMC11210154; doi:10.1186/s12944-024-02169-y)
Supplement: Supplementary file 7 — Supplementary Material 7 [file 12944_2024_2169_MOESM7_ESM.pdf]

LIPIDS For EEDIT & I Thenticate.docx

WORD COUNT	4844	TIME SUBMITTED	13-APR-2024 02:07PM
		PAPER ID	108244961

Abstract

Background: The final decision on fasting or non-fasting for routine lipid profile examination in a standard, healthy population is unclear. Whereas the United States and European protocols state that fasting for regular lipid analysis is unnecessary, the North American and Chinese guidelines still recommend fasting before routine lipid testing. **Aim:** This study aimed to unravel the contradiction between the different protocols of lipid profile testing worldwide and clarify the effect of diet only on doing lipid profile testing in a regular, healthy population. **Methods:** A literature search was run to December 2023. We include in our analysis the studies done from the date 2000 till now because this period appears for the first time the contradiction of guidelines for lipid prophile testing. A planned internal validity evaluation was performed using the National Institute of Health (NIH), the NIH quality measurement means for observational cohort, case-control, controlled interventional, and cross-sectional studies. Data were synthesised according to Rev-Man 5.3. **Results:** Eight studies with a total of 244.665 participants. Six studies' standardised mean difference in cholesterol showed significant differences in overall effect among fasting and non-fasting states (p<0.00001), likewise high-density lipoprotein cholesterol (p<0.00001). At the same time, with triglycerides and Low-density lipoprotein cholesterol, there were notable variations in overall effect between the fasted and non-fasted states (p<0.00001 and p = <0.001, respectively). **Conclusions:** This meta-analysis concluded that fasting for lipid profile

testing is preferred as a conservative model to reduce variability and increase consistency in patients' metabolic status when sampling for lipid testing.

Keywords: Fasting, Non-fasting, Lipid profile testing, Prediction, Healthy population.

1. Introduction

Examining fasting blood lipid levels can offer valuable information about the effects of different diets and metabolic processes. However, it's important to consider whether these levels accurately reflect the impact of individual foods or meals consumed throughout the day.

During 24 hours, the human body remains in a state of non-fasting and absorption for more than 18 hours [1]. In a study conducted by Acevedo-Fani and Singh [2], it was observed that the process of digesting, absorbing, incorporating into the circulatory system, and clearing lipids from different foods and meals is influenced by a range of factors that can be classified into two categories: modifiable and unmodifiable. Factors that cannot be changed include diseases, genetic history, gender, age, and menstrual status.

However, lifestyle choices such as engaging in regular exercise, smoking cigarettes, consuming alcoholic beverages, taking prescription drugs, and making specific food choices are regarded as factors that can be modified. The body's rate of processing lipids is influenced by various factors [3]. In individuals with normal weight and those who are obese, consuming a single meal with a higher total fat content leads to an elevation in the postprandial response of chylomicron triglycerides [4].

[Figure 1]

Although humans typically don't fast or consume less fat regularly, it was previously believed that blood samples for lipid assessment should be taken after 8-12 hours of fasting. This was based on the changes in serum triglycerides during a fat tolerance test. Furthermore, fasting helps to prevent lipemic serum and ensures accurate measurement of low-density lipoprotein (LDL) levels using the commonly used Friedewald's formula in the laboratory [5]. Non-fasting samples have numerous clear advantages:

1) Staying away from the difficulty of prolonged fasting and early morning sampling
2) Minimising the risk of hypoglycemia in diabetic patients
3) A non-fasting state is better for cardiovascular risk prediction, according to guidelines in many countries [6,7]

The highest correlation between peak triglyceride levels assessed four hours after meals and a cardiovascular event has been demonstrated by research [8,9]. Furthermore, there is evidence suggesting a correlation between insulin resistance and levels of lipids or lipoproteins after a meal [10]. In addition, post-meal triglyceride levels that are higher than normal and lower levels of high-density lipoproteins (HDL) cholesterol can be strong indicators of insulin resistance [11]. Community-based studies have shown that consuming food and following non-fasting routines for routine lipid

testing have resulted in minimal changes in lipid profiles that are not clinically significant [6,7,11–15].

The major prospective trials have observed significant mean changes in various lipid parameters. Upon analysis, the recorded changes were as follows: triglycerides showed an increase of 0.3 mmol/L (26 milligrammes/dL), total cholesterol exhibited a decrease of 0.2 mmol/L (8 milligrammes/dL), HDL cholesterol experienced a decrease of 0.1 mmol/L (4 milligrammes/dL), LDL cholesterol displayed a decrease of 0.2 mmol/L (8 milligrammes/dL), calculated remnant cholesterol saw an increase of 0.2 mmol/L (8 milligrammes/dL), and estimated non-HDL cholesterol demonstrated an increase of 0.2 mmol/L (8 milligrammes/dL).

The study revealed that the levels of HDL cholesterol, apolipoprotein A1, apolipoprotein B, and lipoprotein(a) remained unaffected by whether the participants were fasting or non-fasting.

Furthermore, the capacity to forecast cardiovascular disease using both non-fasting and fasting concentrations is similar [6,7,12]. Fasting lipid testing is recommended if the levels of triglycerides exceed 440 mg/dL when not fasting [7,16].

The American Heart Association's (ACC/AHA) recommendations do not call for fasting to estimate the risk of atherosclerotic cardiovascular disease [17]. It is important to remember that performing a fasting lipid profile to evaluate LDL cholesterol levels is recommended. This is especially important for individuals with non-HDL cholesterol

levels below 5.7 millimol/L (220 milligrams/dL) or triglyceride levels above 5.7 millimol/L (500 milligrams/dL). These lipid profiles can be used as possible indicators for inherited and secondary factors contributing to hypertrophy [7].

This study seeks to consolidate the results of previous smaller studies into a comprehensive meta-analysis. The goal is to investigate the potential impact of fasting, non-fasting, or both on lipid profile testing in the general population. Based on our understanding, this study represents a groundbreaking meta-analysis involving a substantial sample size of 244,665 participants. It aims to shed light on the global controversy surrounding this subject.

2. Resources and Procedures

2.1. Methods

2.2. The current systematic review is being reported under the guidelines set by the Preferred Reporting Items for Systematic Reviews and Meta-Analyses (PRISMA) checklist, widely recognised as the standard for reporting systematic reviews [18]. This systematic review adheres to the most recent edition of the Cochrane Handbook for Systematic Reviews of Interventions in its methodology [19]. Additionally, it has been registered on Prospero with the number **CRD42022376871.**

2.3. Data Sources

2.4. This study thoroughly searched various online databases, such as Medline (via PubMed), Scopus, Web of Science, Cochrane, Virtual Health Library (VHL), and Global Index Medicus (GHL), as well as the references of the included studies. Additionally, the study explored related articles up until December 2023.

The study consists of in this analysis the studies done from the date 2000 till now because this period appears for the first time the contradiction of guidelines for lipid prophile testing. Broad search filters were applied to find all the studies by using the following search strategy: ("Lipids" OR ("fatty acids") OR "Ceroids" OR "Fats" OR "Glycerides" OR "Glycolipids" OR" Lipoproteins" OR" Lipopolysaccharides") AND ("Fast* "OR" Fasting" OR ("Hunger Strikes") OR ("Intermittent fasting") OR ("Time-Restricted Feeding")) AND ("Postprandial Periods") OR "non-Fast$" OR" nonFast$" OR" non-fasting "OR ("Postcibal Period") AND ("Normal population") OR ("Healthy volunteers") OR ("Healthy subject"). The search technique used text words and controlled phrases for the normal population's fasting and non-fasting lipid profiles. The studies are included according to the preferred reporting items for systematic reviews and meta-analyses. (See Appendix 1).

2.5. Study Selection

2.5.1. Inclusion Criteria

Studies satisfying the subsequent criteria were contained in the following:

- Study design: all clinical trials or observational studies that measured the lipid profile in fasting and postprandial states.
- Population: A population of individuals aged between 18-75 years old who are in good health. It is essential to establish a baseline by accounting for the influence of various diseases to eliminate any potential variables that could impact the results of lipid profile testing. This will allow us to isolate and analyse the specific effects of diet on the lipid profile testing process.
- Outcome: Studies reporting the demographic and laboratory findings.
- Language: Only studies published in international scientific journals written in English.
- Studies that had enough information for qualitative and quantitative analyses.

2.5.2. Exclusion Criteria

- The researchers did not suggest sufficient data.
- Assessing lipid profile parameters or comparing concentrations of different lipids parameters on unhealthy individuals were omitted.
- Animal research, posters, duplicate papers, or conference papers were not included.

2.6. Screening and Study Selection

The study exported the searched studies to EndNote X9.1 (Clarivate Analytics, https://clarivate.com/) to remove duplicates. Two independent reviewers [HS, AB]

screened all records for eligibility. Eligibility screening was performed in two steps: in the first step, titles and abstracts were screened, and in the second step, full-text articles of the selected abstracts were retrieved and assessed for eligibility. Disagreements were resolved by discussion with a third reviewer. The following PRISMA diagram illustrates the search procedure and details of the study selection in **Figure 2**.

[Figure 2]

2.7. Data extraction

Data about the patients' demographic features, past medical history, clinical presentation, laboratory values, therapies, and clinical outcomes were extracted. Two reviewers, working independently, collected data from a standardised Microsoft Excel spreadsheet. To ensure the accuracy of the retrieved data, an additional reviewer, independent from the previous two, conducted a thorough examination. All instances of dispute were effectively resolved by engaging in thoughtful and constructive debate.

2.8. Evaluation of the included studies' bias risk

The quality of the included studies was assessed using the National Institute of Health (NIH) scale for observational studies.

2.8.1. Assessing risk of bias in individual studies

Two authors (A.B. and H. S.) evaluated the reliability of the studies using the NIH quality assessment tool for various types of research, including observational

cohort, case-control, controlled interventional studies, and cross-sectional studies [20]. This instrument comprises a set of 14 inquiries of various aspects such as sample size, selection process, exposure assessment, and outcome evaluation. Research articles with a score of 9 or more were classified as having good quality, while those scoring between 5 and 8 were deemed to have reasonable quality. Articles with scores ranging from 1 to 4 were categorised as having low quality.

2.8.2. Assessing the risk of bias across studies

The stated results from all the research were thoroughly scrutinised and compared to assess any potential bias in the evaluated trials. This enabled us to detect and eliminate biased reporting of outcomes. Egger and colleagues found that the reliability of detecting publication bias using the funnel plot method falls when there are fewer than ten pooled studies [21].

Data Synthesis and Analysis

Review Manager Software Version 5.3 (Rev-Man 5.3, Copenhagen, The Nordic Cochrane Centre, The Cochrane Collaboration, 2020). Four studies represented mean and standard deviation [5,11,13,14]. Another four studies represented Median and range [15,23,24,25]. For statistical analysis, the studies were inserted by mean and standard deviation, so the data was transformed into mean and standard deviation values according to the method described by McGrath [22].

2.9. Heterogeneity

The evaluation of heterogeneity involved a visual examination of the forest plots to verify the extent of overlap between the 95% confidence intervals of the pooled estimations. The chi-square test was employed to assess heterogeneity, while the I2 test was used to quantify it. The heterogeneity of the outcomes was deemed significant when the p-value exceeded 0.1 and 2>50%. Evidence of heterogeneity in the data for LDL-cholesterol and triglycerides was observed in the study. A random-effects model was employed to address this heterogeneity. Additionally, sensitivity analysis, subgrouping analysis, and prediction interval were calculated to assess the impact of heterogeneity on the study outcomes and determine its magnitude (trivial, moderate, or substantial).

P-values less than 0.05 for overall standardised mean difference (SMD) were considered statistically significant. UN 5 inconsistency (I2), Chi-square (X2), and Tau-square tests were run to check for heterogeneity.

2.9.1. Sensitivity Analysis

For the purpose of evaluating the influence of 5 each study on the overall results, 23 a leave-one-out analysis was conducted to address the variability observed in LDL cholesterol levels. In addition, a specific subgroup analysis was performed for TG. A study that significantly deviated from the norm was excluded to assess the collective effect and accommodate potential variations.

2.9.2. Sub-grouping analysis.

Sub-grouping analysis was conducted based on patients' metabolic status by separating countries into fat-rich and fat-poor meal countries.

2.9.3. Calculation of 95% prediction interval

The summary meta-analysis estimates M, the two-sided crucial t-value t1-0.05/2, k-1, and the standard deviation for the prediction interval SDPI are required to construct the 95 per cent prediction interval. With k being the number of papers included in the meta-analysis, DF=k-1, and probability level 0.025 are used. The SDPI, also known as the standard deviation of the prediction interval, has the formula SDPI = (τ2+SE2), where τ2 is the estimated heterogeneity, and SE denotes the standard error of the SMD. If the SE was not supplied, its estimated value could be calculated by multiplying the separation between the 95% confidence interval for the SMD by 3.92. The 95% confidence prediction interval's bottom and upper boundaries equal M t1-0.05/2, k-1 SDPI.

3. Results

3.1. Details of the included studies

Eight studies were incorporated, with 244,665 participants matched by age and sex. Seven studies (Cartier et al., 2017 [5]; Sidhu and Naugler, 2012 [11]; Yanget al., 2018 [13]; Langston, 2008 [15]; and Umakanth and Ibrahim, 2018 [25]); Liu et al., 2021 [23]; Szternel et al., 2019 [24] reported separate measurements of lipid parameters in fasting and usual diet lifestyle. Schaefer et al., 2001 [14] reported separate measurements of

lipid parameters in fasting and after four hours of a fat-rich meal. All studies that reported different fasting and non-fasting lipid parameter values were incorporated into the meta-analyses for comparison **Table 1**.

[Table 1]

3.2. Characteristics of included studies

Table 2 was constructed to present the data extraction. Four cross-sectional studies were identified; Sidhu & Naugler, 2012 [11]; Langsted et al., 2008 [15], Liu et al., 2021 [23] and Szternel et al., 2019 [24]; the first study [11] involved 209180 subjects representing 46.9 % male and 53.1 % female with a mean age of 52.8 ranging (18-74) and no available data for BMI for those participants; the second study [15] enrolled 33391 subjects representing 47% male and 53 % female with a mean age of 60 ± 9.5 and BMI 26.5 ± 2.5; the third study [23] enrolled 499 participants divided into 51.6 % male and 49.4 % female with mean age 55 ± 13; the fourth one [24] involved 289 participants distributed into 50.9 male and 49.1 female with median age of 48 ± 1.36. Additionally, three cohort studies were detected: Cartier et al., 2017 [5] (In this study, individuals with diabetes were compared to a control group. The control arm was chosen explicitly for examination) involved 1093 subjects, 50.3 % male and 42.5 female, with a mean age of 62.5 ± 10 years old. The study conducted by Yang et al., 2018 [13] involved 41 55% male and 45% female participants, with a mean age of 25.6 ± 6.2 and a BMI of 21.6 ± 6.2.

Umakathand Ibrahim 2018 [25] included 84 participants; 64.28% were male, and 35.71% were female aged 25 to 60. Finally, the RCT study by Schaefer et al. 2001 [14] (In this study compares CVs Vs controls; we only chose the control arm for our study) included 88 subjects, 85% male and 15% female, with a mean age of 62 ± 8.6 and BMI of 26.2 ± 4.2.

[Table 2]

3.3. Quality assessment

The quality of the involved studies was assessed using the NIH scale. Six studies scored 9, 10, 11, 11, 12, 10; Schaefer et al., 2001 [14], Langsted, 2008 [15], Yang et al., 2018 [13], Sidhu and Naugler 2012 [11], Liu et al., 2021 [23] and Szternel et al., 2019 [24] respectively and were considered high-quality, while two studies, Cartier et al., 2017 [5] and Umakanth and Ibrahim, 2018 [25] targeted (score 8) with fair quality **Table 3.**

[Table 3]

A funnel plot is not accurate for the assessment of Publication bias in this study (less than ten studies), so Egger's regression was utilised, revealing significance for publication bias ($p<0.001$). Subsequently, publication bias was assessed using Egger's equation. Based on the refilled and trimmed number of studies in **Table 4**, a renewed search across databases was conducted to identify an additional two studies; Liu (2021)[23], Szternel (2019)[24] to conceal publication bias across our studies**(Figure 3, Table 4).**

[Figure 3 and Table 4]

3.4. Fasting and non-fasting cholesterol and high-density cholesterol difference

As depicted in **Figures 4 and 5**, the estimated mean difference showed significant differences in cholesterol and high-density lipoproteins between fasting and non-fasting (95% CI, -0.03,-0.02) and (95% CI, -0.06,-0.05), respectively. The overall impact evaluation was significant regarding both metrics (*p*, 0.00001). Z-value was 9.93 and 20.05 for cholesterol and high-density lipoproteins, respectively. X2 was 7.45 (*p*=0.38) and 9.29 (*p*=0.23) for testing heterogeneity, respectively. I2 statistics of the cholesterol levels, fasting and non-fasting, and high-density lipoproteins were I2 = 6 and I2 = 25%, respectively, so a fixed-effect model was employed due to the homogeneity observed in the included studies.

[Figure 4]

[Figure 5]

3.5. Fasting and non-fasting triglycerides and low-density cholesterol difference.

As shown in **Figures 6 and 7**, the estimation means difference showed differences in triglycerides and low-density lipoproteins between fasting and non-fasting (95% CI, 0.38, 0.44) and (95% CI, -0.06, -0.09), respectively. For both metrics, the test for the total effect was significant (*p*<0. 00001), and the Z value was 13.04 and 3.92 for triglycerides

and low-density lipoproteins, respectively. For testing heterogeneity, X2 was 102.4(*p*<0.00001), and 24.4(*p*=0.001) respectively. I2 statistics of the TG levels, fasting and non-fasting, and low-density lipoproteins were I2 =93 and I2 = 71%, respectively. A random-effects model was utilised due to the significant heterogeneity observed in the included studies. Sensitivity and subgrouping analyses were conducted, and the prediction interval was discussed.

[Figure 6]

[Figure 7]

3.6. Sensitivity analysis for LDL-chol.

A random-effects model was employed due to significant heterogeneity in the included studies, alongside conducting a sensitivity analysis for LDL-cholesterol. Leave out Cartier, 2017 [5] resolved heterogeneity **Appendix 2.**

By leaving one out a study in each scenario, heterogeneity was not resolved, so subgrouping analysis was conducted based on patients' metabolic status by separating countries into fat-rich meal and fat-poor meal countries, **Appendix 3** of subgrouping analysis resolve heterogeneity (X2=0.57,*p*=0.45, I2=0 %). Also, prediction intervals were discussed.

Discussion

The characteristics of the included studies were detailed, including the study design, participant demographics, and quality assessment scores. Most of the

studies were of high quality, as indicated by their NIH scores. However, two studies were rated as fair quality, emphasising the need to interpret their results carefully.

The analysis uncovered significant cholesterol and high-density lipoprotein levels differences between fasting and non-fasting states, as evidenced by estimated mean differences and corresponding confidence intervals. Heterogeneity testing and model selection were conducted based on the I2 statistics, with a fixed-effect model utilised for homogenous data and a random-effect model for heterogeneous data.

Regarding cholesterol, a significant difference between fasting and non-fasting levels could be seen in the Forest plot. Overall SMD -0.03, 95% confidence interval (CI) (-0.03, -0.02) with *p*-value<0.00001. Regarding heterogeneity, I2 = 6%, and I2 is the percentage of observed variance that reflects actual effect size variations instead of sampling error. The findings align with studies possessing larger sample sizes: Sidhu and Naugler., 2012 [11]; Langsted., 2008 [15] and Liu et al., 2021 [23]. Having a large sample size is crucial in minimising the standard deviation around the mean and, as a result, reducing error. These findings align with previous studies showing the superiority of larger sample sizes over smaller ones. These studies include Cartier et al., 2017 [5], Yang et al., 2018 [13], Schaefer et al., 2001 [14], Umakanth and Ibrahim., 2018 [25] and Szternel et al., 2019 [24].

In addition, the forest plot revealed a notable disparity in HDL levels between individuals who fasted and those who did not. The overall standardised mean

difference is -0.06, with a 95% confidence interval of (-0.06, -0.05) and a p-value of less than 0.00001. Regarding heterogeneity: The I2 value of 42% and p-value of less than 0.12 suggest a relatively small proportion of true effect in the overall observed effect size variances. The study aligns with the findings of several previous researchers as Sidhu and Naugler, 2012 [11]; Langsted., 2008 [15], Liu et al., 2021 [23] and Szternel et al., 2019 [24] and disagrees with Cartier et al., 2017 [5], Yang et al., 2018 [13], Schaefer et al., 2001 [14], Umakanth and Ibrahim., 2018 [25].

The Forest plot exhibited a statistically significant disparity in triglyceride levels when comparing fasting and non-fasting conditions. Overall SMD 0.38, 95% (CI) (0.33, 0.44) and Z value of overall effect 13.04 with p-value <0.00001. i.e., fasting had a significant difference from non-fasting. According to the prediction interval of triglycerides levels were from 0.25 to 1.21; this study expects most levels (moderate effect) to coincide with the respective CI of overall effect (0.28, 0.41), trivial levels with the range of (0.25 to 0.28) and substantial accurate effect levels with the range of (0.41 to 1.21). Both PI and overall CI of triglycerides were on the same positive side of null, i.e., fasting had a significant difference from non-fasting in our study and future studies. All studies' point estimates and 95% CI were in the positive direction of the null line, except for Yang et al., 2018 [13]. In the Schaefer et al., 2001 [15] study, SMD was within the overall, but few values within its 95% (CI) were in the substantial actual effect of PI. In Cartier et al., 2017 [5], SMD and its 95% CI were in the trivial effect of PI. In Langsted,

2008 [15], Umakanth and Ibrahim 2018 [25], Liu et al., 2021 [23] and Szternel et al., 2019 [24], SMD and its 95% CI were the substantial effects of PI. In Yang et al., [13] although 95% CI has crossed the null line to the negative direction, its point estimate value was within the trivial effect of PI.

According to the LDL data analysis, the forest plot showed a significant difference between fasting and non-fasting levels. Overall standardised mean difference -0.06, 95% CI (-0.09, -0.03) and Z value of overall effect 3.92 with *p*-value <0.0001, i.e., non-fasting had a significant difference from fasting as $p<0.05$. In the studies of Cartier et al., 2017 [5], Sidhu and Naugler, 2012 [11], Yang et al., 2018 [13], Schaefer et al., 2001 [14], Langsted, 2008 [15], Umakanth and Ibrahim, 2018 [25] and Liu et al., 2021 [23]; SMD had the negative direction of the null line, with only 95% CI of Yang et al., 2018 [13] Schaefer et al., 2001 [14] and Szternel et al., 2019 [24], were in the positive direction of the null line, i.e., in the substantial effect of PI. In the study of Yang et al., 2018 [13], Sidhu and Naugler., 2012 [11], and secrete Langsted, 2008 [15], SMD and 95% CI were within the overall moderate effect of PI. However, Umakanth and Ibrahim, 2018 [25] showed that SMD and 95% CI were within the trivial effect of PI.

Similarly, differences in triglyceride and low-density lipoprotein levels between fasting and non-fasting states were observed, with significant effects demonstrated through estimated mean differences and heterogeneity testing. A random-effect model was employed due to significant heterogeneity among the included studies,

necessitating sensitivity and subgrouping analyses to explore potential sources of variation.

Hence, most included studies depended on the Fried Wald equation; logically, TG levels in blood were inversely proportional to LDL-cholesterol levels; normal levels of serum TG and LDL-cholesterol ranged from 150-200 mg/dL and < 135 mg/dL, respectively, and that because TG which represents (25%) is not a major component of LDL-chol but cholesterol have a majority which represents 75% of LDL-chol in the fasting state TG is used for energy production so that the levels of total TG decreases & LDL- cholesterol increases and that explained why total TG at the positive side and LDL- cholesterol at the negative side.

The previous results for all lipid profiles matched and explained according to Kovar and Havel, 2002 [26], Nakajima et al., 2011 [27], and Feingold, 2021 [28] who stated that the appearance of chylomicrons in the blood is followed by a rise in very low-density lipoproteins (VLDL) due to competition for lipolysis between VLDL and chylomicrons [26,27]. Postprandial lipemia results from an increase in both intestine-derived chylomicrons and liver-derived VLDL [29]. Capillary endothelial cells have an enzyme called lipoprotein lipase (LPL) on their luminal surface, which binds to chylomicrons and hydrolyses their triglycerides, releasing free fatty acids (FFAs) that may easily pass into cells and be oxidised for energy or re-esterified for storage Cholesterol ester enrichment [30]. ApoB48 and ApoE levels are preserved throughout

converting chylomicrons to chylomicron remnants. The liver is the major organ that removes remnants from the blood; receptors for chylomicron remnants recognise ApoE and uptake that remnants. So, postprandially, VLDL tends to increase more than chylomicrons [27,31]. After six hours, VLDL converted to LDL in circulation. Peristalsis helps pump chyme into the small intestine while you eat. They occur during digestion and can continue for two hours after emptying the stomach. It takes four to five hours for the stomach to empty into the small intestine after a meal [27,32].

The American Heart Association (AHA) guidelines do not recommend fasting protocol for estimating the risk of atherosclerotic cardiovascular diseases. However, AHA only supposes the fasting lipid testing for patients who will undergo statin therapy as well as in cases where the non-HDL cholesterol level is below 5.7 mmol/L (220 mg/dL) or triglycerides are above 5.7 mmol/L (500 mg/dL) to avoid the effect of lipemic serum. Non-fasting and fasting results should be complementary but not exclusive because these could be signs of hereditary and/or secondary causes of hypertriglyceridemia [7,33]. According to the findings of Wilson et al. [34], it has been observed that the identification of potentially actionable abnormal lipid test results, explicitly fasting triglycerides (TG) levels equal to or exceeding 500 mg/dL, necessitates the reporting of such cases as hypertriglyceridemia. Enhancing the proper utilisation and accurate documentation of lipid tests is expected to enhance their efficacy in the comprehensive care of individuals with a heightened susceptibility to atherosclerotic

cardiovascular disease (ASCVD) occurrences. On a laboratory basis, if the lipemic serum is detected, fasting for 8-12 hours for triglycerides and LDL testing is mandatory; besides, LDL should be technically measured using diagnostic kits, not Friedewald's formula. That is because lipemia affects the Calculation of LDL-cholesterol, and chylomicrons affect measurements of triglycerides.

Specifically, sensitivity analysis for low-density lipoprotein cholesterol involved leaving out individual studies to assess their impact on heterogeneity. Subgrouping analysis based on patients' metabolic status and dietary habits was also conducted to explore sources of heterogeneity further and refine our findings. According to the current statistical data, most lipid measurements, including cholesterol, HDL cholesterol, lipoprotein triglycerides, and LDL, showed significant changes between fasting and non-fasting testing protocols [35].

4. Study strengths and limitations

Firstly, this study identified eight studies involving 244,665 participants, matched by age and sex. These studies reported separate measurements of lipid parameters in fasting and non-fasting conditions, with one study also examining lipid levels after consuming a fat-rich meal; including these studies allowed for a comprehensive analysis of the differences in lipid profiles between fasting and non-fasting states.

As a scientific researcher would do in the initial stages, it is important to note that the smaller trials did not show any variation between fasting and non-fasting.

However, the larger study with a bigger sample size revealed a significant difference, which aligns with our findings.

The study has two limitations: a restricted number of included studies due to stringent inclusion and exclusion criteria and significant heterogeneity observed among studies regarding triglycerides and LDL-cholesterol.

5. Conclusion

As a conservative model to reduce variability and increase consistency in patients' metabolic status at the time of sampling for lipid testing, this meta-analysis favourably leans towards fasting for lipid profile testing; however, significant differences were detected in the measurements.

The study sheds light on how fasting and non-fasting states impact lipid profile testing in the general population. The meta-analysis carries significant implications for clinical practice and patient care.

This study was found that fasting status strongly influences lipid profile data, with notable changes observed in cholesterol, HDL, triglycerides, and LDL levels between fasting and non-fasting states. These findings emphasise the importance of fasting status in clinical lipid profile interpretation and cardiovascular risk assessment. Understanding fasting and non-fasting lipid levels can also help customise patient therapy. Clinicians should know that non-fasting lipid profiles may affect therapy decisions, especially in metabolic illnesses like diabetes, where lipid control prevents

cardiovascular consequences. the study also shows the need for fasting status-specific lipid profile testing guidelines. Doctors should consider recent food intake and patient characteristics when reading lipid tests and making clinical judgements.

This finding shows that fasting and non-fasting lipid profile testing are clinically relevant and emphasises the need for personalised cholesterol therapy that considers fasting status and patient demands. By applying these findings, clinical practitioners can improve cardiovascular risk assessment and patient treatment.

LIPIDS For EEDIT & I Thenticate.docx

ORIGINALITY REPORT

10%
SIMILARITY INDEX

PRIMARY SOURCES

1	academic.oup.com<br>Internet	80 words — 2%
2	www.ncbi.nlm.nih.gov<br>Internet	76 words — 1%
3	lipidworld.biomedcentral.com<br>Internet	47 words — 1%
4	www.nature.com<br>Internet	43 words — 1%
5	Ramy Abdelnaby, Yousef Tarek Sonbol, Khaled T. Dardeer, Anas ELgenidy et al. "Could Osteopontin be a useful biomarker in the diagnosis and severity assessment of osteoarthritis? A systematic review and meta-analysis of recent evidence", Clinical Immunology, 2022<br>Crossref	26 words — 1%
6	www.frontiersin.org<br>Internet	24 words — < 1%
7	www.hindawi.com<br>Internet	24 words — < 1%
8	www.cureus.com<br>Internet	20 words — < 1%

9	Barzilai, Eran. "Alone with Your Thoughts: The Emotional and Behavioral Effects of Explicit and Implicit Social Rejection in Individuals with Suicidal Ideation", The New School, 2022<br>ProQuest	18 words — < 1%
10	www.researchgate.net<br>Internet	16 words — < 1%
11	mdpi-res.com<br>Internet	13 words — < 1%
12	Vivencio Barrios, Carlos Escobar. "Rosuvastatin along the cardiovascular continuum: from JUPITER to AURORA", Expert Review of Cardiovascular Therapy, 2014<br>Crossref	12 words — < 1%
13	assets.researchsquare.com<br>Internet	10 words — < 1%
14	journals.lww.com<br>Internet	10 words — < 1%
15	web-lighthouse.prod.aplaceformom.com<br>Internet	10 words — < 1%
16	www.koreascience.or.kr<br>Internet	10 words — < 1%
17	www.science.gov<br>Internet	10 words — < 1%
18	www2.mdpi.com<br>Internet	10 words — < 1%

19	Ellen T.H.C. Smeets, Ronald P. Mensink, Peter J. Joris. "Effects of tree nut and groundnut consumption compared with those of l-arginine supplementation on fasting and postprandial flow-mediated vasodilation: Meta-analysis of human randomized controlled trials", Clinical Nutrition, 2020<br>Crossref	9 words — < 1%
20	www.grafiati.com<br>Internet	9 words — < 1%
21	Ahmed Elgebaly, Bassant Abdelazeim, Omar Mattar, Mohamed Gadelkarim, Rehab Salah, Ahmed Negida. "Meta-analysis of the safety and efficacy of droxidopa for neurogenic orthostatic hypotension", Clinical Autonomic Research, 2016<br>Crossref	8 words — < 1%
22	Faun, Chang Lin. "Effects of Dietary Fats and Carbohydrate on Insulin Secretion, Inflammation and Gastro-Intestinal Peptides in Abdominally Obese Individuals: A Randomized Controlled Trial", University of Malaya (Malaysia), 2023<br>ProQuest	8 words — < 1%
23	Palmer, Sarah N.. "Equity Sensitivity and Work Outcomes: A Meta-Analysis", Saint Louis University, 2022<br>ProQuest	8 words — < 1%
24	clok.uclan.ac.uk<br>Internet	8 words — < 1%
25	cris.maastrichtuniversity.nl<br>Internet	8 words — < 1%
26	worldwidescience.org	

Internet

8 words — < 1%

27 www.medrxiv.org
Internet

8 words — < 1%

28 " : Clinical Summaries ", Circulation, 2014
Crossref

6 words — < 1%

EXCLUDE QUOTES	OFF	EXCLUDE SOURCES	OFF
EXCLUDE BIBLIOGRAPHY	ON	EXCLUDE MATCHES	OFF
